# Development of a Flexible Microneedle Array Electrode with a High Signal-to-Noise Ratio for Surface Bioelectrical Signal Recording

**DOI:** 10.3390/bios16020108

**Published:** 2026-02-07

**Authors:** Bo Jiang, Ye Wang, Ruiqing Li, Yan Zhou, Lihua Ma, Dingjie Suo, Guangying Pei

**Affiliations:** 1 School of Medical Technology, Beijing Institute of Technology, Beijing 100081, China; 2 School of Mechatronical Engineering, Beijing Institute of Technology, Beijing 100081, China; 3 Beijing Key Laboratory of Brain-Inspired Neural Engineering, School of Medical Technology, Beijing Institute of Technology, Beijing 100081, China

**Keywords:** flexible microneedle electrode, impedance, bioelectrical signal, metal 3D printing

## Abstract

Microneedle array (MNA) electrodes have garnered significant attention for their capacity to record high-fidelity surface bioelectrical signals over extended periods and convenience. However, accuracy limitations in 3D-printed metal MNA electrodes, particularly concerning surface roughness and insufficient tip sharpness, have been reported. Additionally, the prevalent use of nonporous metal substrates often results in poor flexibility. This study proposes a novel MNA electrode featuring a lightweight flexible substrate and sharp, smooth microneedles. Utilizing micron-level metal 3D printing with 316L stainless steel, we fabricated the electrodes in a single step. We evaluated the MNA electrode-skin interface impedance via frequency sweep and assessed mechanical properties using porcine skin, followed by the collection and analysis of bioelectrical signals. The results demonstrate that the contact impedance of the MNA electrode is comparable to that of standard gold cup electrodes, with validated flexibility and strength. Furthermore, the MNA electrodes achieved a high signal-to-noise ratio and minimal motion artifacts during recording, thereby enhancing both comfort and signal quality. The efficient production process facilitates the broader application of metal MNA electrodes.

## 1. Introduction

Surface bioelectrical signals are often regarded as important auxiliary tools for clinical diagnosis. By collecting and analyzing bioelectrical signals such as electroencephalogram (EEG), electrocardiogram (ECG), and electromyogram (EMG), it is possible to effectively assess physical functions and mental states; these signals serve as key indicators for evaluating treatment efficacy [1,2,3]. Consequently, electrodes capable of capturing accurate and reliable electrophysiological signals are integral to biosensors and medical devices. Currently, the gold standard for detecting EEG signals involves the use of wet electrodes combined with a conductive paste [4]. However, the preparation process is cumbersome, and the electrodes tend to dry out over time, leading to an increase in the electrode-to-skin interface impedance (EII), which subsequently affects signal quality [5,6,7]. In applications such as brain–computer interfaces and EMG-controlled prosthetics, electrodes often need to be worn for extended periods, making traditional wet electrodes unsuitable for long-term use. To address the need for prolonged signal recording, dry electrodes that do not require conductive gels have been developed, offering a quicker installation process. However, owing to the presence of the high-resistance stratum corneum (SC), dry electrodes frequently exhibit high and unstable EII. Additionally, their performance may be compromised by factors such as excessive size and motion artifacts [8,9,10,11,12].

**Figure 1:**
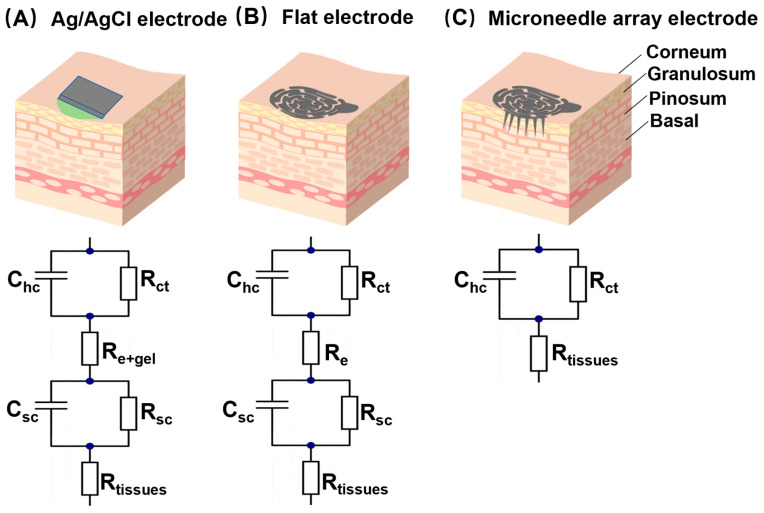
Schematic representation and equivalent electrical model for (**A**) conventional wet electrodes (the green-colored substance is conductive paste), (**B**) flat electrodes, and (**C**) microneedle array electrodes.

Microneedle array (MNA) electrodes have attracted increasing attention. An MNA electrode can effectively penetrate the high-impedance SC without touching the dermis or deeper tissues, thus avoiding significant pain [13,14,15]. By embedding an anchoring mechanism in the superficial dermis, the surface contact between the electrode and the skin is transformed into an internal anchoring contact, fundamentally reducing relative displacement. Simultaneously, by utilizing mechanical properties that match the biomechanics of the skin, movement stress is buffered, maintaining the stability of the interface impedance [16,17]. Figure 1 illustrates equivalent circuit models for three electrode types, which are composed of resistors (R) and capacitors (C) in parallel/series combinations to approximate skin electrical impedance. Here, hc is the half-cell potential between the electrode and skin; C_hc_ is the charge transfer equivalent capacitance, R_ct_ is the charge transfer resistance; R_e+gel_ and R_e_ are the series resistances of sweat/moisture or sweat/moisture + electrolyte gel/cream between the electrode and the skin. R_sc_ and C_sc_ are the equivalent resistance and capacitance of the epidermis layer; R_tissue_ is the resistance of the dermis and underlying tissues. With the growing demand for real-time detection, particularly for measuring biological signals in home monitoring devices, MNA electrodes are well-suited for the development of wearable sensors because of their miniaturization, rapid response, and low detection limits. Recent studies have indicated that MNA electrodes have facilitated continuous monitoring, multifaceted analysis, and even intelligent therapy. For example, Kim et al. introduced a curved MNA electrode that exhibited improved sweat resistance [18]. Wang et al. fabricated a polydimethylsiloxane MNA coated with layers of titanium and gold, resulting in lower EII and enhanced signal quality during ECG monitoring [19]. Additionally, Om et al. developed flexible conductive fabric MNA electrodes that support continuous, long-term, and high-quality ECG/EMG monitoring [17].

A variety of preparation methods for MNA electrodes have been reported [20,21,22,23,24,25]. While typical lithography techniques are highly accurate, they are also expensive and environmentally unfriendly [26]. Additionally, MNA electrodes produced via laser cutting often exhibit a rough surface [27]. 3D printing has emerged as a novel manufacturing technology capable of directly generating complex shapes that are challenging to achieve with other processes [28]. However, MNA electrodes with heights of less than 1 mm and tip diameters of less than 50 microns exceed the technical limitations of conventional metal 3D printing devices, making single-step molding unfeasible [29]. Presently, most MNA electrodes utilize materials such as silicon, metals, and polymers. Silicon offers good biocompatibility and high hardness, facilitating penetration of the SC; however, its lack of flexibility poses a risk of breakage of the electrodes within skin [30]. Metal materials, such as stainless steel, provide excellent corrosion resistance and mechanical strength, reducing the likelihood of breakage. However, the complexity of their structures presents significant challenges due to equipment accuracy limitations, and their nonporous substrate lacks flexibility [29,31]. Regarding polymers, electrodes made from a silver-coated elastomer, such as polydimethylsiloxane, may bend under applied pressure [32]. Material selection is crucial for long-term applications. The use of a flexible substrate enhances conformal contact with human skin, thereby minimizing motion-induced artifacts [33,34]. While ensuring the strength of microneedles is essential, there is an increasing demand to improve the elasticity of the substrate. A novel material based on p-xylene has been developed, but its preparation process involves several complex steps, including thermal oxidation, reactive ion etching, Parylene film deposition, stripping technology, and sputtering. Such complex, labor-intensive fabrication processes that lack automation can limit the large-scale economic production of MNA electrodes [35] and hinder their ability to penetrate tougher skin. Although numerous methods exist for preparing flexible MNA electrodes, achieving satisfactory performance through simple and practical techniques remains a significant challenge.

One issue arises when the flexible substrate is composed of the same material as the MNA electrode: it typically either lacks the required flexibility or is too soft to effectively penetrate the skin. Conversely, the use of different materials introduces challenges related to adhesion. Additionally, the tradeoff between high hardness and fracture toughness poses a considerable problem. For example, materials such as silicon and SU-8 have high hardness characteristics but are brittle, which increases the risk of structural fracture and compromises safety performance. Furthermore, these materials must satisfy the requirements of nontoxicity and good biocompatibility.

In this study, an MNA electrode featuring a specialized structural design is constructed from medical-grade 316L stainless steel. The substrate exhibits good flexibility, a compact size, and lightweight properties, allowing it to be securely affixed with medical tape. Furthermore, we employ advanced metal 3D printing technology, which achieves high precision at the micron level, in contrast to the millimeter-level accuracy that is typically found on the market. This technology enables the one-step manufacturing of MNA electrodes. To the best of our knowledge, there are no existing research reports on metal additive manufacturing technology, specifically Micro Laser Powder Bed Fusion (MLPBF) for one-step 3D printing of MNA electrodes. To evaluate the performance of this MNA electrode, we conducted several human experiments in which three different types of electrodes were compared. We measured the EII, and recorded the characteristics and signal-to-noise ratios (SNR) of the EEG, ECG, and EMG signals to comprehensively assess the performance. Overall, the excellent performance of the designed MNA electrode is demonstrated by its sound structural design and high-precision printing.

## 2. Materials and Methods

### 2.1. Structural Design and Manufacture of 3D-Printed Electrodes

The needle body and substrate of the MNA electrode are constructed from medical-grade 316L stainless steel, which attains an elastic modulus of approximately 200 GPa. This material exhibits excellent biocompatibility, as well as high strength and high hardness, making it well-suited for the design of MNA electrodes [36,37,38]. Furthermore, it possesses good electrical conductivity, allowing for effective transmission of electrical signals without the need for a conductive layer, such as a gold coating, thereby reducing costs. Additionally, 316L stainless steel demonstrates remarkable corrosion resistance, enabling it to withstand the corrosive effects of various biological fluids and thereby extending the service life of the MNA electrode.

**Figure 2:**
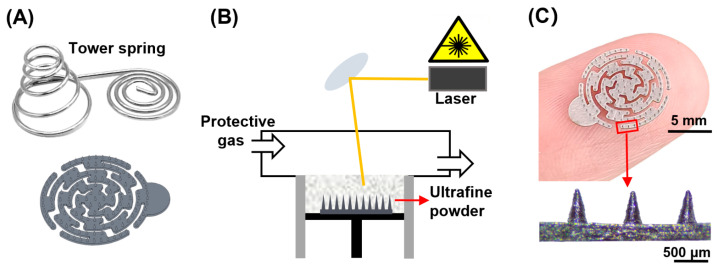
MNA electrode design and manufacture. (**A**) Tower spring inspired model. (**B**) MLPBF micrometer metal 3D printing. (**C**) Real photo and local magnification of an MNA electrode under a microscope.

To enhance the compatibility of MNA electrodes with the skin, the substrate must possess a certain degree of flexibility. The tower spring structure can deform under both compressive and tensile loads, making it effective for shock absorption, cushioning, and support [39]. In this study, we drew inspiration from the tower spring structure. Ultimately, the electrode was designed as a circular structure composed of a multi-stage rotating cantilever beam featuring a serpentine cutout pattern, as illustrated in Figure 2A. The underlying design employs a specialized hollow shape to reduce material continuity by incorporating specific holes within the electrode material, which alters the mass distribution and mitigates stress concentration effects. Under external force, the hollow regions are more susceptible to deformation [40]. Furthermore, by adjusting the thickness of the substrate, we can further decrease the stiffness and increase the flexibility.

The MNA electrode model was designed using computer-aided design (AutoCAD 2021) software (San Rafael, CA, USA). The MNA electrode model comprises an array of 115 conical needles, each with a microneedle height of 500 μm, a base diameter of 200 μm, and an aspect ratio of 5:2. The tip-to-tip spacing is 800 μm, and the entire needle array is situated on a substrate measuring 10 mm in diameter and 0.25 mm in thickness. It features a circular labyrinth cutout design, which includes a semicircle with a radius of 2 mm at the edge of the substrate, facilitating the connection of an electrical signal transmission wire, which is linked to a metal button at the opposite end. This information was subsequently imported into a P100 ultraprecision metal printer (Aixway, Suzhou, China).

The MNA electrode was printed using 316L stainless steel powder in an inert gas-protected environment, where the inert gas was high-purity argon, and the oxygen content was maintained below 100 ppm. Upon completion of the printing process, the microneedle parts were then immersed in a 75% alcohol solution for ultrasonic cleaning and disinfection, with a cleaning duration of 4 h. This was followed by an additional 10 min of ultrasonic cleaning with deionized water, and the process was repeated at least twice to ensure thorough cleaning.

For the MNA electrode studied in this paper, a state-of-the-art commercial MLPBF process was utilized, in which a smaller laser spot and a thinner layer thickness were incorporated to further refine the grain morphology of the material, thereby enhancing its performance. The printing accuracy reaches or exceeds 2 microns, with a surface roughness Ra value of no greater than 0.8 microns. Figure 2B presents an overview of the printing process, while Figure 2C illustrates the actual MNA electrode. The entire needle body is well-proportioned and smooth. The diameter of the tip after printing is measured as approximately 30 μm; these tips are sufficiently sharp to effectively puncture the skin and fulfill the intended function.

### 2.2. Mechanical Strength and Skin Penetration Test of the MNA Electrode

Fresh pork was purchased from the market, and after being thoroughly cleaned, the meat was separated from the skin using a blade. The pig skin was then cut to the required size for the experiment. The experiments were not conducted directly on live pigs. Specifically, the skin was cut into 60 mm × 60 mm square specimens (≈10 mm thick) using a surgical scalpel. Next, the pig skin was fixed with clamps. The prepared MNA electrode was placed on it and subjected to appropriate thumb pressure for approximately 3 s before being pulled out. This process was repeated 100 times by repositioning the MNA electrode and reapplying pressure. Finally, a microscope (Murzider, Dongguan, China) was used to observe the changes in the microneedle morphology and background of the electrode before and after insertion.

Pig skin was rinsed with clean water and then cut into 20 mm × 20 mm square specimens (≈10 mm thick) using a medical surgical blade. The MNA electrode was subsequently inserted into the pig skin and secured with a cable tie. Next, the entire sample was immersed in a formalin solution. After three days of treatment, the sample was removed, rinsed with water, and uniformly coated with a refrigerant on the surface before being placed in a freezer set to −30 °C. After half an hour, the sample was removed and sliced using a cryotome (CM1950, Nussloch, Germany) with the section thickness set to 25 μm. The pig skin slices, following puncture, were observed with an inverted fluorescence microscope (Eclipse Ti-E, Nikon, Tokyo, Japan) at room temperature (20 °C).

### 2.3. Successful Penetration of the MNA into Skin and Skin Compatibility Test

Pig skin was collected and cleaned with water, after which the specimen was cut into a 20 mm × 20 mm square with a thickness of approximately 10 mm using a surgical scalpel. A force of 5 N was applied to the electrode and maintained for 5 s before being released. The MNA electrode was subsequently removed after insertion into the skin. Methylene blue dye (1% *w*/*v*) was then applied to the skin and left on for 2 h prior to cleaning. The dye was carefully removed from the skin using Kimtech wipes soaked in acetone; any resulting blue mark indicated successful rupture of the SC. The penetration rate was calculated via Equation (1).

(1) $$\%Penetration\;=\;\frac{N_{p}}{N_{T}}\;\times 100\;$$

The MNA electrode was initially affixed to the forehead of a 29-year-old healthy male volunteer via medical tape, followed by the application of an elastic band to maintain an appropriate pressure. After one hour, the MNA electrode was removed, and the skin condition was evaluated and photographed every ten minutes until recovery.

### 2.4. MNA Electrode Flexibility Test

The flexibility evaluation of the MNA electrode comprised both finite element model simulation and physical testing. The finite element model was developed using Abaqus (Abaqus 2018, Dassault Systèmes, Vélizy-Villacoublay, France) with the following key parameters: the mass density of the stainless steel was set at 7980 kg/m^3^, the Young’s modulus was set at 193 GPa, and the Poisson’s ratio was 0.3. Regarding meshing, a tetrahedral configuration was utilized. Boundary conditions were set such that the four outermost edges of the microneedles were secured, while a vertical force of 1 N was applied downward. In the physical testing of the MNA electrode, the electrode was fixed to a support, and a force of 1 N was applied at its center by metal tweezers to observe the deformation of the substrate. Concurrently, the maximum displacement at the center was measured with a Vernier caliper. After the pressure was released, the recovery of the deformed MNA electrode was assessed.

### 2.5. Impedance Measurement Experiment

The impedance from electrode to skin to electrode was measured using an E4990A impedance analyzer (Keysight Technologies, Santa Rosa, CA, USA). The test was conducted over a frequency sweep range of 20 Hz to 2 kHz. Three different types of electrodes were compared in this experiment: the developed MNA electrode, the clinical standard gel electrode (brain circulation crescent electrode), and the gold cup electrode provided by Shanghai Litu Medical Appliances Co., Ltd. (Shanghai, China). A medical conductive paste produced by Suzhou Locai Medical Technology Co., LTD (Suzhou, China). Prior was used with the gold cup electrode. The gold cup electrode is a rigid electrode with a diameter of 10 mm and a thickness of 0.8 mm, made of copper with an extremely thin gold plating (<10 μm). During normal signal acquisition, it does not undergo plastic deformation, and the elastic deformation is also minimal and almost imperceptible. The MNA electrode was connected to the instrument via a soldered wire extending from the back. The area of skin that would be in contact with the electrode was cleaned and disinfected with 75% alcohol, and the MNA electrode was affixed to the center of the subjects’ foreheads via medical tape, spaced 5 cm apart. Given that the MNA electrode is more sensitive to pressure, subjects wore an elastic band on their foreheads to apply an appropriate pressure. During the experiment, the subjects completed the test while seated, and the procedure was repeated using the gel electrode and the gold cup electrode. The impedance of each electrode was calculated according to Equation (2), where *Z*_1_ represents the positive electrode-to-skin impedance and *Z*_2_ denotes the negative electrode-to-skin impedance. A total of 10 healthy subjects (8 males and 2 females, aged 26 ± 3 years) were recruited for this study. All participants provided written informed consent in accordance with the guidelines of the Declaration of Helsinki. This study was approved by the Ethics Committee of the Beijing Institute of Technology (BIT-EC-H-2022150).

(2) $$Z_{electrode}\;=\;\frac{Z_{1}\;+\;Z_{2}}{2}\;$$

### 2.6. EEG Recording

Five healthy subjects (5 males, aged 26 ± 3 years) were recruited for this study. All participants provided informed consent in accordance with the guidelines of the Declaration of Helsinki. This study was approved by the Ethics Committee of the Beijing Institute of Technology (BIT-EC-H-2022150). EEG measurements were conducted via a unipolar connection method. The working electrode was positioned at the standard location of the 10–20 system (Fp1), the reference electrode was placed on the left mastoid (TP9), and the ground electrode was positioned at the center of the forehead. The MNA electrode was secured with medical tape; as it is sensitive to pressure, an appropriate pressure that the subject could tolerate was applied via an elastic band. The gold cup electrode was also held in place with medical tape, while the gel electrode was directly affixed to the corresponding site. EEG signals were recorded with a commercial amplifier (Neuroscan SynAmps2 EEG amplifier, Compumedics Neuroscan, Charlotte, NC, USA) at a sampling rate of 500 Hz. During the eyes-open state, participants focused on a cross displayed on a screen, and EEG data were collected for three minutes. The participants subsequently closed their eyes for another three minutes. Additionally, one subject was selected for blink signal collection, with the electrode still positioned at Fp1. The subject was instructed to blink once per second during a two-minute blink test, during which signals from all three types of electrodes were collected. The *RMS* values of the signal and noise data for all segments extracted from each EEG signal were computed to represent the average amplitude. The *RMS* value was determined according to Equation (3), where *N* denotes the total number of data points and *X_n_* represents the n-the data point. The *SNR* was calculated via Equation (3).

(3) $$\begin{array}{l} RMS\;=\;\sqrt{\frac{1}{N}\sum_{n=1}^{N}{\left|X_{n}\right|}^{2}}\\ SNR\;=\;20log_{10}\frac{RMS_{signal}}{RMS_{noise}}\; \end{array}$$

### 2.7. ECG Recording

A wearable wireless ECG recording device was developed. The external dimensions of the ECG equipment formed a cuboid module measuring 6.5 cm in length, 5.5 cm in width, and 3 cm in height. For the microcontroller unit, the STM32F405RGT6 chip from STMicroelectronics (Geneva, Switzerland), which is a high-performance 32-bit microcontroller, was used. The device also incorporated the application-specific integrated circuit chip ADS1292R from Texas Instruments (Dallas, TX, USA) and a rechargeable lithium-ion battery with an output voltage of 5 V (Shenzhen Zhongshunxin Technology Co., Ltd., Shenzhen, China).

The electrodes were positioned at the first intercostal line of the middle clavicle along the left margin of the sternum and on the left and right inferior abdomen. If necessary, gentle pressure may be applied with the fingers to enhance the adhesion of the electrodes to the skin. Five healthy subjects (5 males, aged 26 ± 3 years) were recruited for this study. We measured the ECG signals of the subjects in a sedentary state, as well as the dynamic ECG signals while they walked at a speed of approximately 1.2 m/s. The SNR of the signals recorded with the three different types of electrodes was calculated as follows: First, the recorded signal was segmented into 15 single heartbeat cycles, each containing the P, Q, R, S, and T waves. Using the R peak as a reference, these 15 cycles were aligned in time so that the average heartbeat cycle could be obtained by calculating the average of the data points at each time point across the 15 cycles. This average signal was then subtracted from each original heartbeat cycle to extract noise. The *RMS* values for different periods were then used to calculate *A_noise_*, that is, defined as the noise *RMS* value measured over the entire time interval consisting of 15 periods. The mean value was then subtracted from the average signal to calculate the average signal *RMS* value from the resulting unbiased signal and further determine the SNR. The calculation of the SNR was based on Equation (4). Single-factor analysis of variance (ANOVA) was performed using SPSS version 26.0 (SPSS, Inc., Chicago, IL, USA).

(4) $$SNR\;=\;20log(\frac{A_{Average\;signal}}{A_{noise}})$$

### 2.8. sEMG Recording

Five healthy male subjects, aged 26 ± 3 years, were recruited for this study. Three types of electrodes were used to record the EMG signals of the subjects’ right biceps. The MNA electrode and gold cup electrode were secured with medical tape, while the gel electrode was directly affixed to the skin surface. Electrodes were placed 4 cm apart on the sterilized biceps, with the ground electrode positioned laterally. The three types of electrodes were connected to a commercial physiological signal recording and analysis system (MP160, BIOPAC Systems, Inc., Goleta, CA, USA). The sampling rate was set to 1 kHz, and the test duration was 120 s. Prior to the test, the subjects were instructed to drop and relax their arms to collect resting-state data for 5 s. Subsequently, they were required to raise the dumbbell (2 kg), position their forearm at a 90° angle to the upper arm, and hold this position for 10 s before lowering the dumbbell. After this process was complete, the MNA electrode was replaced with a standard gel electrode and a gold cup electrode, and the aforementioned steps were repeated. The *RMS* and *SNR* values for both the signal and noise were calculated, with the SNR derived through filtering and analysis of the acquired signals. The relevant calculation formulas can be found in Equation (3).

A 50 Hz harmonic infinite impulse response (IIR) comb digital filter was employed to eliminate power frequency interference. Following this, signal and noise segments were extracted from the filtered data. In the recorded dataset, a 300-millisecond segment from the middle of the sEMG waveform corresponding to each peak triggered by muscle activity was designated the signal segment. Conversely, a 600-millisecond segment from the resting-state portion between two sEMG waveforms was classified as the noise segment. The *RMS* values of both the signal and noise data were calculated for each EMG signal to reflect their average amplitudes, and the SNR of the EMG signal was subsequently determined. Here, the RMS signal and RMS noise refer to the *RMS* values in the signal and noise regions, respectively, obtained during the muscle contraction and relaxation phases. Single-factor ANOVA was performed with SPSS version 26.0 software.

## 3. Results and Discussion

### 3.1. Mechanical Properties and Biocompatibility

**Figure 3:**
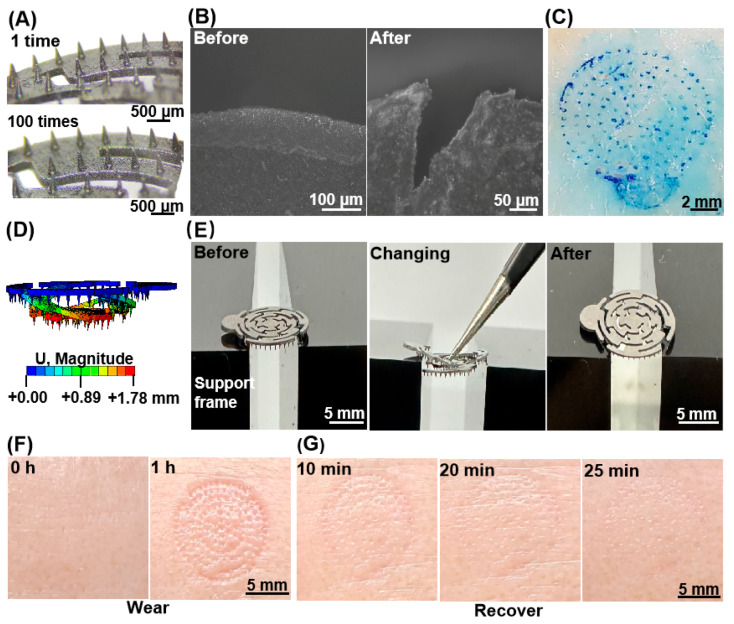
Mechanical characterization, safety, and biocompatibility tests. (**A**) Comparison before and after MNA insertion into pig skin 100 times (strength test). (**B**) Pig skin sample insertion depth test. (**C**) Penetration test of pig skin samples. (**D**) MNA substrate flexibility simulation test. (**E**) MNA entity flexibility entity test. (**F**,**G**) Human skin compatibility tests.

To verify that the mechanical strength of the MNA electrodes meets the safety requirements, pig skin, which is anatomically similar to human skin and possesses comparable mechanical properties [41], was utilized as the test material. In the experiment, thumb pressure significantly exceeding the normal load (approximately 5 N) was applied to the prepared metal MNA electrode [42]. Changes in the microneedle body before and after the experiment were observed with a microscope, as illustrated in Figure 3A.

The microneedle structure remained intact after puncturing the pig skin once and 100 times. Throughout the test procedure, no plastic deformation or fracture occurred in either the microneedle body or the substrate. These findings indicate that 316L stainless steel, which was used as the MNA electrode material, meets the mechanical strength requirements, effectively minimizing the risk of needle collapse.

To verify that the depth of microneedle penetration into skin is within a safe range, we utilized fresh pig skin as an alternative to human skin for testing. Figure 3B shows the comparison of pig skin tissue sections before and after puncture with the MNA electrode. Following the puncture, an inverted triangular cavity structure resembling the cross-section of the MNA electrode is visible on the surface, with a hole depth of approximately 200 μm, which is approximately 2/5 of the height of the MNA electrode. This observation suggests that the microneedles did not fully penetrate the skin, likely because of the inherent elasticity of the skin. Compared with previous reports [14], our design effectively penetrates the high-impedance SC and reaches the conductive living epidermis, without extending into the dermis, which contains nerve endings. Consequently, the microneedle length complies with safety requirements.

To evaluate the penetration rate of the MNA electrode into the skin, pig skin was again used as a model to replace human skin in the experiment. We used 1% *w*/*v* methylene blue dye, which is a surgical dye with a high protein affinity and is usually used to evaluate the penetration effect of MNA electrodes [17]. Methylene blue can bind to epidermal tissue, but does not interact with the hydrophobic cuticle layer, so it only stays in the epidermis and the inner layer of the dermis. After the dye is removed from the skin surface, a residual blue mark indicates that the cuticle layer has been successfully pierced. As shown in Figure 3C, out of the 115 MNA microneedles fabricated, 113 successfully penetrated the skin, achieving a penetration rate of 98.3%. This result indicates that almost all the microneedles effectively pierced the cuticle layer.

To evaluate the flexibility of the MNA electrode substrate, a finite element simulation was conducted to assess the displacement of the electrode model. Based on previous reports regarding the interaction between an MNA electrode and skin, a three-dimensional superelastic and anisotropic prestressed multilayer material must be constructed to represent the skin model. This model is quite complex, and simulating the deformation with all microneedles inserted into the skin would result in an excessively large computational demand. Consequently, a simplified MNA electrode model simulation was employed in this study. Owing to the absence of skin support, a force of 1N was applied after multiple simulations. The simulation results, depicted in Figure 3D, indicate a maximum displacement of 1.78 mm. A deformation test was subsequently performed on the printed object, as illustrated in Figure 3E. When a force of approximately 1N is applied to the center of the microneedle array via tweezers, deformation results similar to those of the simulation are obtained, with a maximum displacement of 1.86 mm. Upon withdrawal of the applied force, the MNA electrode returned to its original shape, demonstrating that the MNA electrode exhibits good flexibility even under low forces.

To evaluate the compatibility of the MNA electrode with human skin, we conducted a continuous test on the forehead of a volunteer for one hour. Immediately following the test, the MNA electrode was removed, and the skin condition was assessed. As illustrated in Figure 3F, a distinct imprint is evident on the skin immediately after MNA electrode removal. However, there is no indication of inflammation or redness; the elastic deformation of the skin gradually recovers, and the mark on the surface diminishes. As shown in Figure 3G, the imprint is nearly completely resolved within 30 min. Throughout the entire procedure, the subjects reported no pain or discomfort. These findings suggest that 316L stainless steel, when used as an MNA electrode, causes minimal damage to the skin and demonstrates good compatibility.

### 3.2. Impedance Measurement

**Figure 4:**
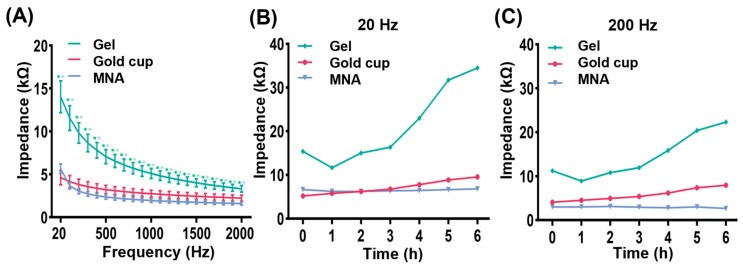
Gel electrode, gold cup electrode and MNA electrode impedance results. (**A**) Changes in electrode impedance with frequency (error bars: standard error, *n* = 10. ** *p* < 0.01). (**B**) Changes in electrode impedance with time at 20 Hz (*n* = 1). (**C**) Changes in electrode impedance with time at 200 Hz (*n* = 1) (*n* represents the number of subjects).

The impedance signal between a bioelectrode and skin serves as a crucial measure for evaluating electrode performance. Figure 4A shows that the EII values of the three compared electrodes progressively decrease as the input current frequency increases. Notably, the EII of the gel electrode is the highest, while the EII of the MNA electrode consistently remains lower than that of the gel electrode across the frequency range of up to 2000 Hz. A significant difference in EII was observed between the MNA electrode and the gel electrode (*p* = 0.001). Furthermore, the impedance of the MNA electrode is comparable to that of the gold cup electrode. The measured EII comprises both resistive and capacitive components, which decrease with increasing frequency.

To assess the stability of the MNA electrode, we conducted a long-term wear test in which the MNA electrode was continuously worn on the skin of a volunteer for 6 h, and the EII was measured hourly to evaluate the long-term performance. As illustrated in Figure 4B, the EII of the MNA electrode demonstrates greater stability throughout the test period than those of the gold cup electrode and the gel electrode at 20 Hz. Concurrently, the EII values of the gel electrode and the gold cup electrode tend to increase over time. Notably, after 2 h, the EII of the gold cup electrode surpasses that of the MNA electrode, whereas the EII of the MNA electrode remains relatively constant. Furthermore, we also examined the EII changes for various electrodes over time at a low frequency of 200 Hz; the results are presented in Figure 4C. The MNA electrode exhibits the lowest impedance and the smallest amplitude variation, indicating superior stability. In contrast, the EII values of the gold cup electrode and the gel electrode generally increase over time.

### 3.3. EEG Recordings and SNR Analysis

**Figure 5:**
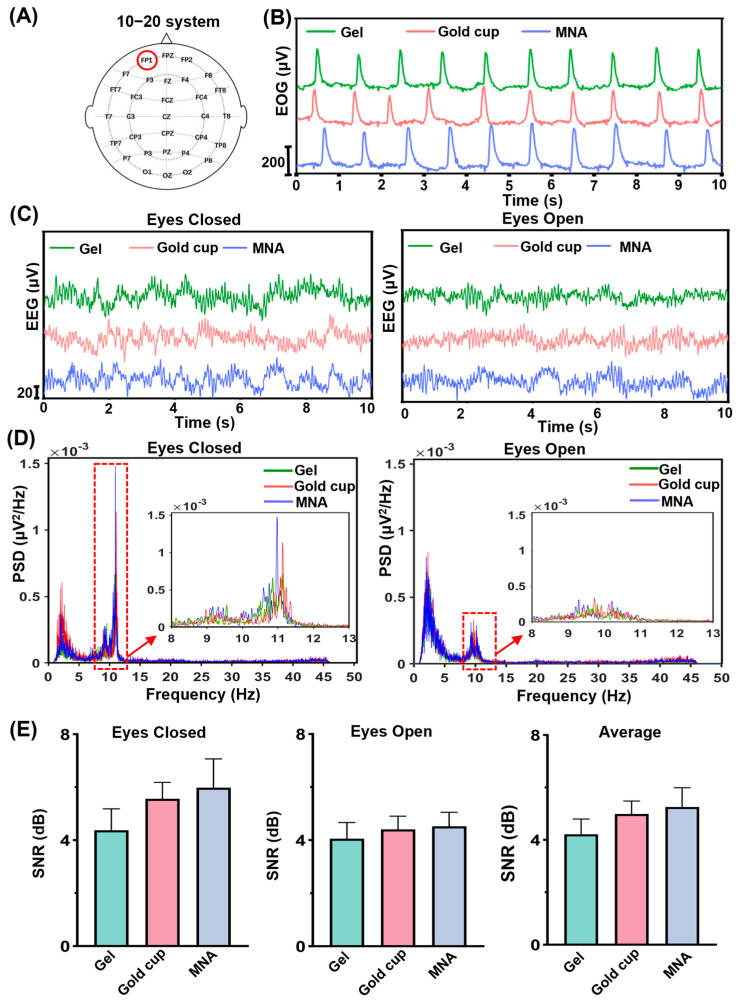
EEG signals recorded by gel electrodes, gold cup electrodes and MNA electrodes. (**A**) Wearing positions for EEG and EOG recording. (**B**) Three electrodes were used to record the eye electrical waveform during blinking in the Fp1 channel region (*n* = 1). (**C**) Frontal lobe (Fp1 channel) electrical EEG signals were measured for eyes closed and eyes open (*n* = 1). (**D**) Frequency analysis of frontal EEG signals (PSD: power spectral density). (**E**) EEG SNR comparison (error bars: standard error, *n* = 5).

As shown in Figure 5A, the electrode was positioned at the Fp1 location. We tested the blink signal at 1 Hz and recorded the EEG signals with the eyes in both the open and closed states. The blink signal results are presented in Figure 5B and demonstrate that the 1 Hz electrooculogram (EOG) signal can be successfully captured by all three types of electrodes. Concurrently, single-channel EEG signals were recorded while the eyes were resting in both the open and closed positions. Figure 5C displays the original EEG signals acquired via gel electrodes, gold cup electrodes, and MNA electrodes. In many relevant studies, the α-wave rhythm is frequently utilized as a key indicator for assessing the quality of EEG signals under various states of eyes-open and eyes-closed states [43,44]. Figure 5D shows that the α-wave rhythm is significantly observable with all three types of electrodes in the closed-eye state but is considerably suppressed when the eyes are open. Among the signals the MNA electrode α-wave rhythm is the most pronounced, and its frequency analysis results align with the waveform characteristics. Upon further analysis, as depicted in Figure 5E, the MNA electrode displayed the highest mean SNR of all electrode types, though the difference between electrode types was not found to be statistically significant (*p* > 0.05). The data collected during the resting state with the eyes open and closed indicate that the MNA electrode has the highest SNR, followed by the gold cup electrode, whereas the gel electrode has the lowest SNR.

### 3.4. ECG Recordings and SNR Analysis

**Figure 6:**
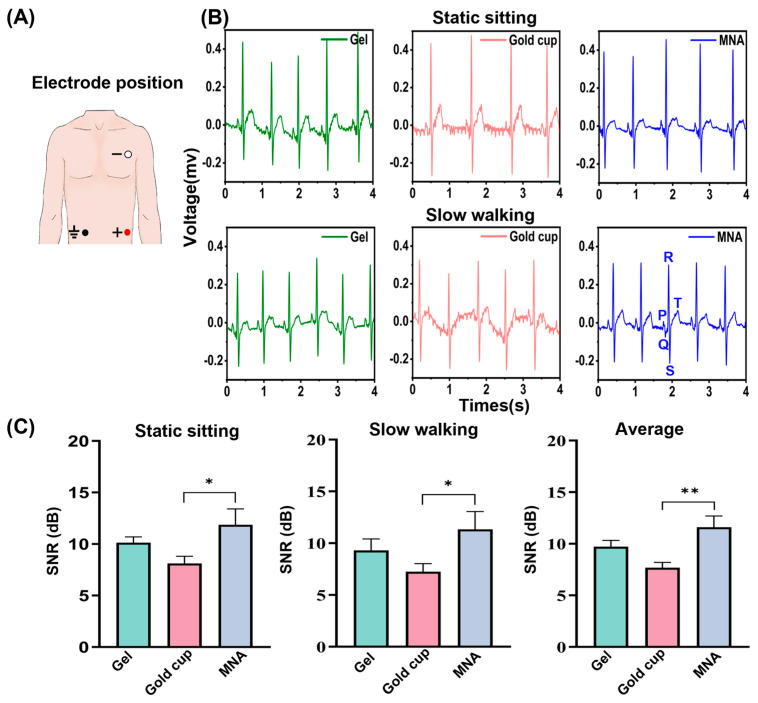
ECG signals recorded by the gel electrode, gold cup electrode and MNA electrode. (**A**) Electrode wearing positions for ECG recording. (**B**) ECG signals during static sitting and slow walking. (**C**) ECG SNR comparison (error bars: standard error, *n* = 5. * *p* < 0.05, ** *p* < 0.01).

To evaluate the performance of different types of electrodes in ECG detection, two states were considered, sitting and slow walking, during which typical ECG signals were collected. Figure 6A,B presents a comparison of the signals obtained from the three different types of electrodes under static conditions, specifically when the subject is seated in a chair. The key features of the P wave, QRS complex, and T wave are clearly captured and visible. Figure 6C shows that there are significant differences (*p* = 0.040) in the SNR between the MNA electrode and the gold cup electrode in the sitting and slow walking states. Furthermore, the experimental findings reveal that under static conditions, the overall noise level of the gold cup electrode is greater, and the signals collected by the gold cup electrode are lower signal quality than those obtained by the MNA electrode and the gel electrode. This discrepancy may be attributed to the sliding of the contact interface caused by the subject’s breathing when the gold cup electrode makes contact the skin through the conductive paste, resulting in artifacts that diminish the signal recognition capability [45].

Under dynamic conditions, when subjects walked in a straight line along a corridor at a speed of approximately 1.2 m/s, several major ECG waveforms from the electrode remained observable. However, both the gel and gold cup electrodes exhibited a certain degree of baseline drift, as illustrated in Figure 6B. Among them, the baseline drift of the gold cup electrode is the most pronounced, and it also displays significant noise, resulting in a relatively unclear waveform. The P wave, Q wave, and T wave are particularly difficult to distinguish because of this issue, which is likely caused by the subjects’ arm movements while walking. The drift and distortion of the ECG signal obtained with the MNA electrode caused by the subject’s arm movement and swing can be virtually ignored, and the P wave, QRS complex, and T wave can all be clearly distinguished. The baseline drift and distortion caused by movement are minimized. Figure 6C shows that in the motion state, there is a significant difference in the SNR between the MNA electrode and the gold cup electrode (*p* = 0.024). The average SNR shows a significant difference (*p* = 0.001) between the MNA electrode and the gold cup electrode, with the MNA electrode exhibiting the best overall performance. Although all the types of electrodes are affected by noise and baseline drift during body movement, they perform optimally in a resting state, where their baseline drift is significantly lower than that in motion. The MNA electrode, in particular, displays a smaller baseline drift, likely due to its ability to maintain a more stable contact interface after insertion into the SC of the skin, thereby mitigating some interference caused by motion artifacts. The experiment shows that the MNA electrode has high sensitivity for detecting ECG signals.

### 3.5. Surface EMG (sEMG) Recordings and SNR Analysis

**Figure 7:**
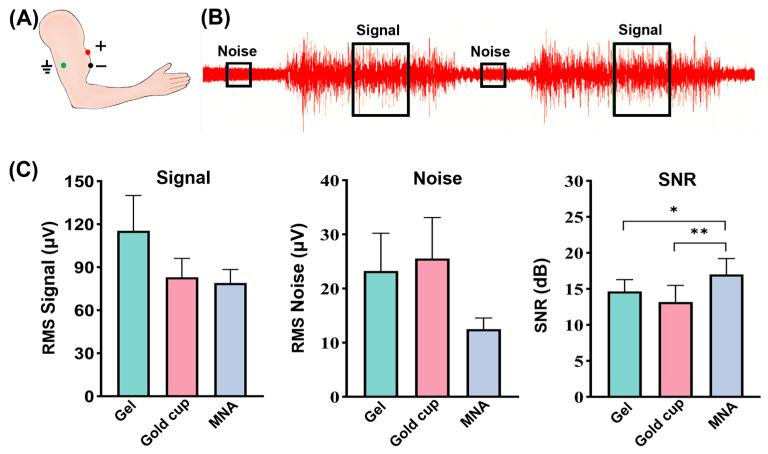
sEMG signals recorded by the gel electrode, gold cup electrode and MNA electrode. (**A**) Electrode wearing positions for ECG recording. (**B**) Schematic of the extraction of signal and noise segments for SNR analysis. (**C**) RMS signal, RMS noise, SNR comparison (error bars: standard error, *n* = 5. * *p* < 0.05, ** *p* < 0.01).

To investigate the performance of the electrodes in measuring muscle activity, as presented in Figure 7A, we recorded the sEMG signals of the biceps. The measurement results indicate that all types of electrodes can accurately reflect the contraction and relaxation of the biceps muscle. Figure 7B shows that during relaxation, when no force is exerted, the sEMG signal remains at a low level. Conversely, when the muscle contracts, the internally generated electrical signals are rapidly activated, resulting in a swift increase in the signal amplitude. The signal and noise segments were selected, and the root mean square (RMS) and SNR were calculated. According to Figure 7C, the SNR was analyzed. As shown in the statistical results, the SNR of the MNA showed a significant difference (*p* = 0.043) from that of the gel electrode, and a significant difference (*p* = 0.001) from that of the gold cup electrode. The results show that the MNA electrode achieved higher SNR performance, proving the superiority of the MNA electrode in biopotential recording once again.

During muscle movement, the tissue exhibits complex geometry and dynamic fluctuations, which pose challenges for conventional flat and rigid electrodes in adapting to the skin texture. This results in unstable contact between the skin and the electrode. Furthermore, although the gold cup and gel electrodes have good stability at the electrode–skin interface owing to their extensive coverage and the use of strong bonding materials such as foam, during muscle contraction, traction and resistance can introduce interference, thereby reducing the SNR [46]. In contrast, the MNA electrode directly penetrates through the SC with its tiny needle-like structure, achieving more stable electrical contact with the deeper layers of the skin, which results in a higher and more stable SNR.

## 4. Conclusions

This study successfully developed a novel MNA electrode through structural optimization strategies, enhancing its flexibility to better conform to the curvature of the skin surface. Additionally, high-precision micron-level metal 3D printing technology was employed to fabricate 316L stainless steel MNA electrodes in a single step. The resulting electrodes exhibit a uniform shape, smooth surface, and sharp needle tips. Importantly, it maintains excellent conductivity without the need for a conductive coating. They produce stable and consistent signals, enabling long-term signal recording while demonstrating superior biological signal monitoring performance. They effectively reduce motion artifacts during ambulation and achieve a high signal-to-noise ratio. This work offers new structural insights for the advancement of flexible microneedle electrodes and overcomes the limitations of previous metal 3D printing methods that could not produce sharp and smooth microneedle electrodes in a single step. This method can save time and labor costs, enhance production efficiency, and facilitate mass production. Nonetheless, while the 316L stainless steel material utilized in this research possesses a degree of flexibility, it may not be as flexible as some specialized materials, and its conductivity is not optimal. Future research should explore alternative microneedle electrode materials, such as nickel-titanium alloys, which exhibit unique shape memory effects and superelasticity, as well as the use of flexible nanocomposite materials with enhanced conductivity. Furthermore, performance and comfort could be improved by optimizing coatings and adjusting needle density to further enhance functionality. This study primarily focuses on the design of a single array and does not include a comparative analysis with other reported multi-electrode disks or smaller-sized anti-deformation arrays. Such comparisons could further elucidate our findings regarding mechanical and electrical performance. The sample size used for core biological signal validation is relatively small (*n* = 5). However, future work requires a larger subject population to enhance statistical power, particularly for metrics with non-significant differences (e.g., EEG signal-to-noise ratio). The current coverage of action scenarios is insufficient; future studies need to include actions such as bending over and sit–stand transitions to thoroughly validate the anti-interference capability of the MNA electrodes.

## Data Availability

The data that support the findings of this study are available on request from the corresponding author but restrictions apply to the availability of these data, which were used under license for the current study, and so are not publicly available.
